# A systems biology network analysis of root resorption biomarkers and their clinical relevance in orthodontics: a pilot study

**DOI:** 10.1186/s44342-026-00079-2

**Published:** 2026-09-28

**Authors:** Parzhin Moradi, Vahid Mollabashi, Amir Taherkhani, Homa Farhadifard, Erfan Ayubi

**Affiliations:** 1 Department of Orthodontics, School of Dentistry, Hamadan University of Medical Sciences, Hamadan, Iran; 2 Department of Orthodontics, School of Dentistry, Hamadan Dental Research Center, Hamadan University of Medical Sciences, Hamadan, Iran; 3 Research Center for Molecular Medicine, Institute of Cancer, Avicenna Health Research Institute, Hamadan University of Medical Sciences, Hamadan, Iran; 4 Social Determinants of Health Research Center, Institute of Health Sciences and Technologies, Hamadan University of Medical Sciences, Hamadan, Iran

**Keywords:** Orthodontic root resorption, Systems biology, Hub genes, Therapeutic targets, Biomarkers

## Abstract

**Background:**

External apical root resorption is a frequently reported iatrogenic consequence of orthodontic tooth movement. This pilot study used an integrated systems biology approach to identify signaling pathways, key genes, and candidate salivary biomarkers associated with orthodontic root resorption to support future functional research.

**Methods:**

The present study used a sequential workflow that began with identifying root resorption-associated genes from a systematic literature review and the DisGeNET database. Subsequently, a protein–protein interaction (PPI) network was constructed to identify hub genes, followed by pathway enrichment and Gene Ontology analyses. Next, a Gene Regulatory Network was built to predict upstream transcription factors. Finally, an ELISA assay was performed on saliva samples from 12 patients with orthodontic root resorption and 12 matched controls to validate the computational findings experimentally. The diagnostic utility of the selected markers was assessed using receiver operating characteristic (ROC) curves.

**Results:**

A set of 160 genes was found to be linked to the process of orthodontic root resorption. Within the PPIN, 13 genes were recognized as central hub genes. Among these, CASP1 and SPP1 were selected for the ELISA assay. Gene Regulatory Network analysis computationally identified IRF4 as the most impactful upstream regulator of CASP1 and SPP1. The ELISA analysis confirmed overexpression of salivary Caspase-1 and Osteopontin proteins in patients with orthodontic root resorption compared with those without signs of the condition. Caspase-1 emerged as a strong candidate for non-invasive diagnosis of root resorption in orthodontic patients, with impressive efficacy (AUC = 0.9861; Youden Index = 0.917).

**Conclusion:**

This study suggests that elevated salivary levels of CASP1 and SPP1 are associated with radiographically confirmed OIIRR. These proteins represent candidate biomarkers whose potential biological relevance warrants longitudinal evaluation, rather than definitive predictive tools or therapeutic targets. Therefore, given the preliminary nature of these bioinformatics analyses and the limited sample size in the clinical validation, further large-scale prospective studies are required to confirm their diagnostic and therapeutic utility.

**Graphical Abstract:**

**Figure:**
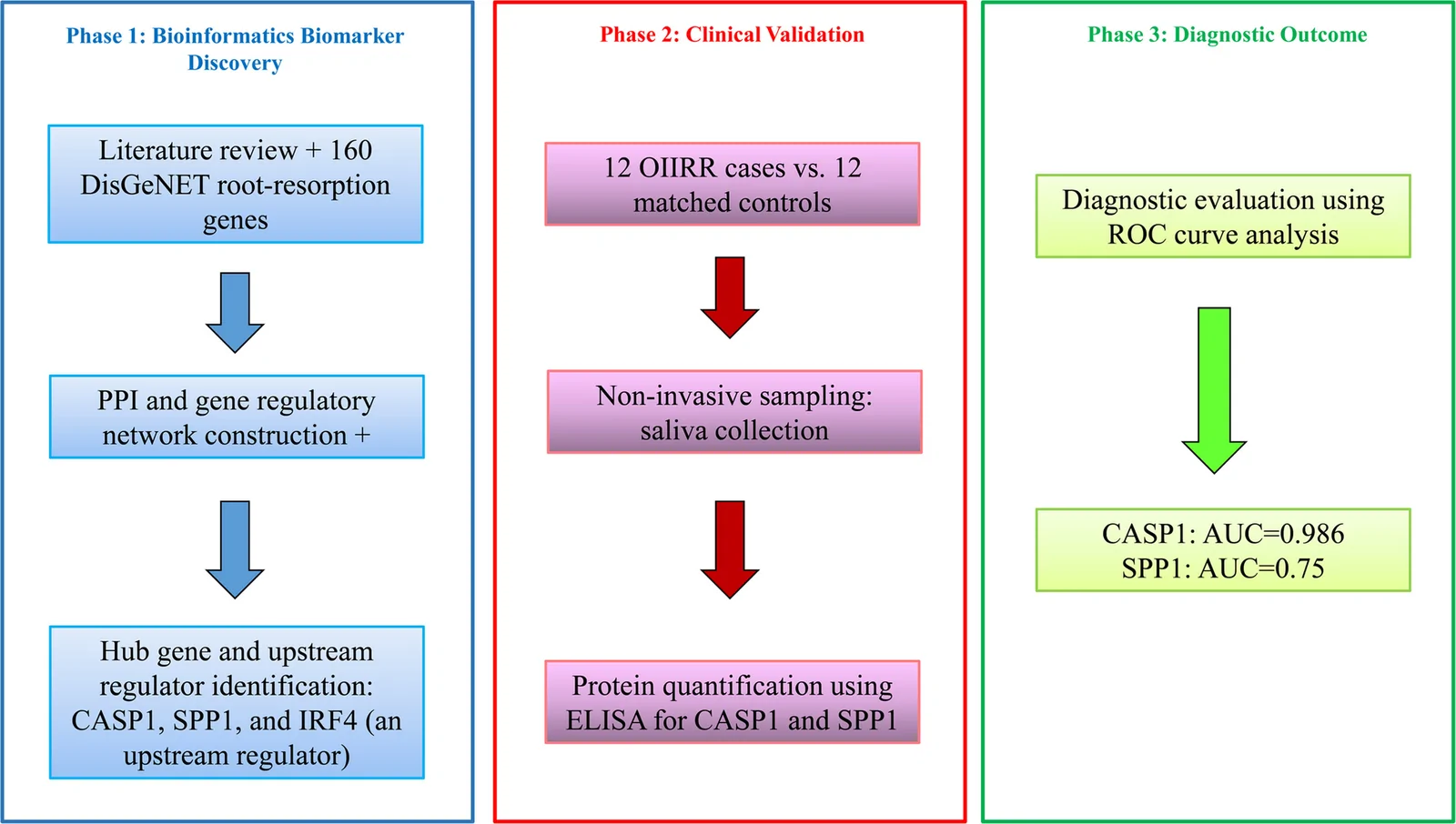

**Supplementary Information:**

The online version contains supplementary material available at https://doi.org/10.1186/s44342-026-00079-2.

## Introduction

Orthodontic tooth movement is a dynamic biological process that requires a delicate balance between bone formation and resorption. However, this process carries an inherent risk: orthodontically induced inflammatory root resorption (OIIRR). OIIRR is a common and unavoidable consequence of orthodontic treatment, characterized by the loss of mineralized cementum and dentin [1]. Orthodontic force triggers a sequential cellular response that begins with removal of the hyalinized zone. This exposure leads to osteoclast-mediated resorption of the root surface, facilitating tooth movement. Simultaneously, osteoblasts contribute to bone remodeling at the margins, and resorbed root areas are gradually replaced by normal bone once the hyalinized tissue is eliminated or the force is reduced [2].

Root resorption is a multifactorial phenomenon influenced by systemic factors (e.g., genetics), local conditions (e.g., periodontal or periapical disease, trauma), and iatrogenic factors like orthodontic treatment. Mechanical factors such as treatment duration, force magnitude, and root displacement further increase the risk of apical resorption [3]. Severe root resorption can lead to serious complications such as tooth mobility or even permanent tooth loss [4]. In cases where teeth already have short roots, extraction may become necessary [2, 5]. Root resorption is typically classified as mild, moderate, or severe [6, 7]. A more detailed grading system defines Grade I as irregular root surface changes, Grade II as root shortening of less than 2 mm, Grade III as resorption between 2 mm and one-third of the root length, and Grade IV as resorption exceeding one-third of the root length [8]. Histological changes in humans are usually detectable 3–5 weeks after orthodontic force initiation [4, 6, 9]. While the radiographic examination is the most common clinical detection method, apical resorption often becomes radiographically evident only after 5–6 months [6, 10]. Studies indicate that nearly all orthodontically treated patients experience some degree of root shortening, though it is usually mild and clinically insignificant. However, some patients may exhibit severe resorption, defined as a loss exceeding one-third of root length [6]. Reports show that approximately 93% of adolescents undergoing orthodontic treatment develop some form of root resorption [2, 11], with 12–17% experiencing moderate to severe apical resorption [2]. Notably, severe apical resorption (> 3 mm) occurs in 10–20% of cases [2, 12, 13]. While all permanent teeth can undergo resorption [14], incisors are the most susceptible, whereas molars have the lowest risk [6, 15].

Accumulating evidence indicates that genetic factors strongly influence individual susceptibility to orthodontically induced inflammatory root resorption. Previous studies have demonstrated significant associations between root resorption severity and genetic variants involved in inflammatory signaling, immune regulation, and bone remodeling pathways, including genes within the Interleukin-1 signaling cascade, purinergic receptors, and inflammasome-related mediators. Also, evidence indicates that genetic polymorphisms in *IL1B* [16], *IL-6* [17], and *P2RX7* [18] are involved in the etiology of root resorption [19]. These findings suggest that host-specific molecular responses to orthodontic force play a decisive role in determining resorption outcomes, beyond mechanical variables alone. These findings suggest that host-specific molecular responses to orthodontic force play a critical role in determining resorption outcomes, beyond mechanical variables alone. However, a significant gap remains in translating these genetic and molecular insights into non-invasive, clinically applicable diagnostic tools. Most previous investigations have relied on polymorphism analyses or tissue-level observations, which do not offer real-time monitoring capabilities during active orthodontic treatment.

**Fig. 1:**
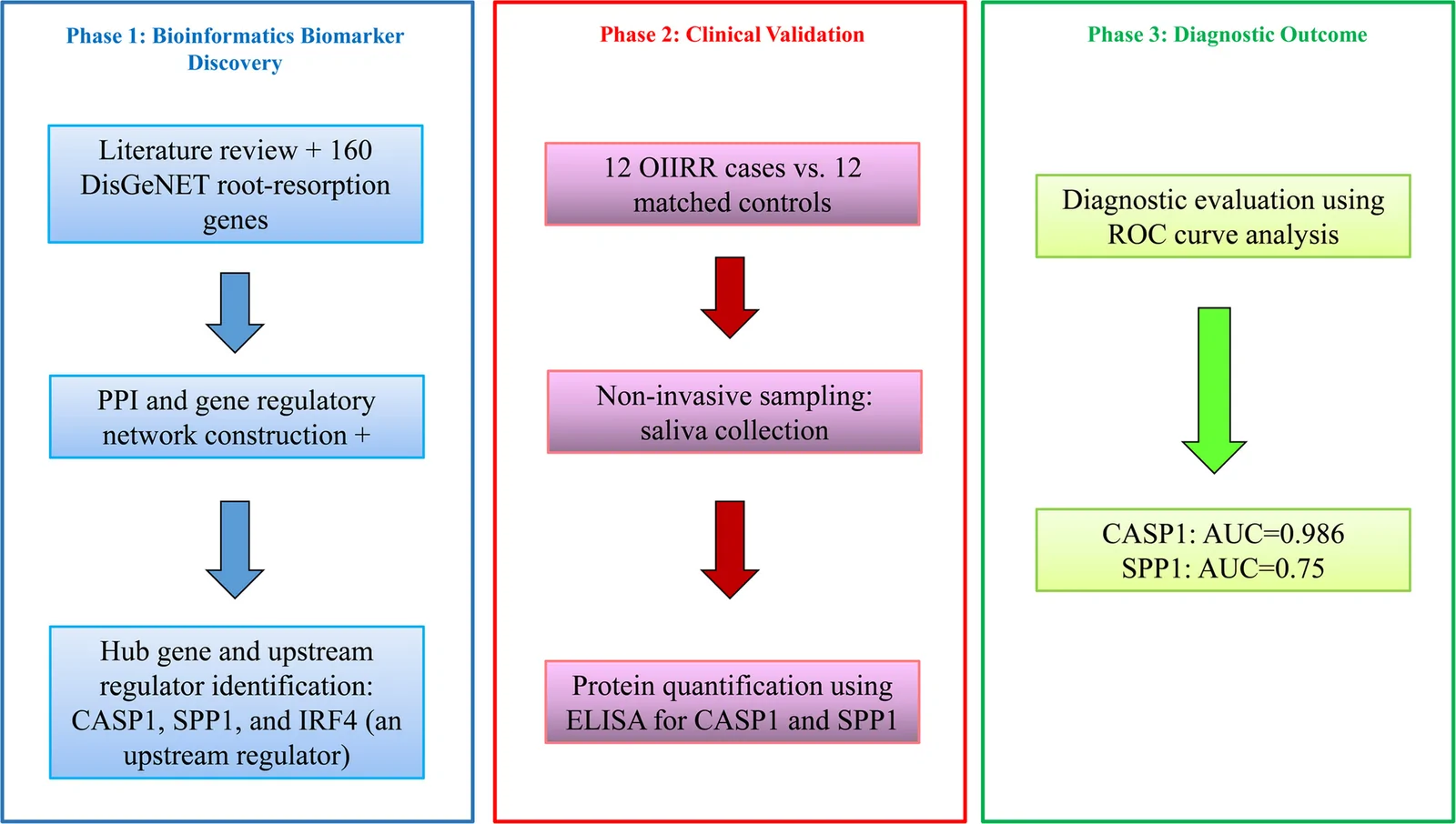
Overall workflow of the study

We hypothesized that key genes involved in inflammation and bone remodeling, which are critical to the etiology of root resorption, are reflected downstream at the protein level in saliva. Consequently, these salivary proteins may show distinct expression profiles in patients with OIIRR compared with unaffected individuals. Therefore, the primary objective of this study was to identify central molecular mediators of root resorption using an in silico network-based approach and subsequently to evaluate their clinical utility as non-invasive salivary biomarkers. Figure 1 presents an overview of the study design.

## Materials and methods

The investigation was structured into two separate components: (A) computational bioinformatics analysis and (B) laboratory-based experimental validation. The methodology for each element is outlined individually below.

### (A) Bioinformatics analysis

#### Data collection

To identify candidate genes associated with root resorption, a comprehensive search was conducted in PubMed/MEDLINE, Web of Science, and Scopus. The search strategy combined MeSH terms and keywords using the following keywords: “root resorption,” “orthodontically induced root resorption,” “biomarker,” “proteome,” “protein,” “gene,” and “genome.”

Peer-reviewed original and reviewed studies, including bioinformatics, clinical, in vivo, and in vitro investigations, were eligible if they reported significant changes in gene or protein expression related to mechanically induced root resorption. Animal studies and conference abstracts were excluded. After deduplication and independent screening by two reviewers, eligible candidate genes were extracted and integrated with root resorption-associated genes obtained from the DisGeNET database.

Gene symbols and protein aliases were standardized and mapped to official human gene nomenclature using the HUGO Gene Nomenclature Committee (HGNC) database.

In parallel, DisGeNET was used as a complementary resource to retrieve gene–disease associations related to root resorption. DisGeNET integrates evidence from curated databases, genome-wide association studies, experimental models, and the scientific literature. Root resorption-associated genes were extracted using the DisGeNET disease identifier C0035851. To retain a sufficiently broad gene set while ensuring a minimum level of literature-supported evidence for the condition, all retrieved genes were filtered using a baseline Gene–Disease Association (GDA) score threshold of > 0.01. Genes obtained from DisGeNET were merged with those identified in the literature review, and duplicate entries were removed before constructing the PPI network.

Two independent authors screened the literature and extracted gene information based on predefined relevance criteria.

#### Construction of protein interaction networks and identifying meaningful clusters and central genes

Protein–protein interactions (PPIs) associated with root resorption were first identified with the online STRING tool (v12.0) (STRING, available at https://string-db.org/), database version 12.0. The network was refined by removing all unconnected nodes. This PPI Network was then visualized and analyzed for its structural properties in Cytoscape (v3.10.3), available at www.cytoscape.org.

In Cytoscape, the Molecular Complex Detection (MCODE) plugin was used to identify highly interconnected clusters within the PPI Network, representing groups of proteins potentially involved in common biological processes or pathways. MCODE analysis was performed using the following parameters: degree cutoff = 2, node score cutoff = 0.2, k-core score = 2, and maximum depth = 100. Clusters were considered significant if they had an MCODE score greater than three and contained at least 10 nodes.

The network analyzer tool then computed the degree, betweenness, and closeness centrality for each node. Hubs were subsequently defined as nodes with degree and betweenness centrality values at least twice the network average, and a closeness centrality score exceeding the mean.

#### Functional gene annotation and pathway enrichment analyses

Pathway enrichment and Gene Ontology (GO) annotation analyses were performed using the g:Profiler web-based platform (https://biit.cs.ut.ee/gprofiler/gost). These analyses encompassed three key components: biological process (BP), cellular component (CC), and molecular function (MF) enrichment evaluations. For pathway enrichment and BP annotation analyses, only significant modules were analyzed, whereas the entire gene set involved in root resorption was used for CC and MF identification. Statistical significance was determined using established threshold parameters: a false discovery rate (FDR) below 0.05 and a minimum of 10 enriched genes per term.

#### GRN construction

This study constructed a Gene Regulatory Network (GRN) incorporating hub genes and transcription factors (TFs). The iRegulon plugin was employed to identify candidate TFs for two hub genes that demonstrated significant involvement in root resorption pathogenesis and whose expression profiles had been confirmed in saliva samples from patients with root resorption using an ELISA test. For this analysis, TFs exhibiting a normalized enrichment score (NES) exceeding 10 were deemed statistically significant.

### (B) Wet-lab (experimental) validation

This study is reported in accordance with the Strengthening the Reporting of Observational Studies in Epidemiology (STROBE) guidelines for case-control studies.

#### Study design

This clinical investigation was conducted as a matched cross-sectional case–control study. A total of 24 patients undergoing orthodontic treatment were included 12 with radiographically confirmed orthodontic root resorption (case group) and 12 without evidence of root resorption (control group), matched for age and gender. Matching was frequency-based and the independent t-test remains appropriate. All participants received treatment with the MBT appliance system with a 0.022-inch slot.

Participants were recruited consecutively from patients receiving orthodontic treatment at the Department of Orthodontics, Hamadan University of Medical Sciences, Hamadan, Iran. Clinical records and routine radiographs were screened to identify eligible patients meeting the predefined inclusion and exclusion criteria. Patients were approached during routine follow-up visits and invited to participate.

Root resorption status was determined at a single time point using radiographic records obtained during routine orthodontic follow-up, either during active treatment (after a minimum of 10 months) or at treatment completion. No longitudinal follow-up or intervention was performed after group allocation.

Group allocation (case or control) was determined before saliva sampling based on radiographic findings. Saliva collection and ELISA analysis were performed after group classification, ensuring biomarker assessment was blinded to orthodontic treatment planning decisions.

#### Inclusion and exclusion criteria

Eligible participants in both groups were undergoing orthodontic treatment based on extraction protocols, had at least 24 natural teeth, and had no cavitated caries. Written informed consent was obtained from all participants before enrollment.

To be included in the case group, patients were required to have radiographic evidence of external apical root resorption, with no more than three years between the initiation of orthodontic treatment and the diagnosis of root resorption. Control group participants exhibited no radiographic signs of root resorption on panoramic images.

Participants were excluded if they had systemic diseases, a history of allergies, were smokers, or had taken antibiotics within three months before saliva sampling. Additionally, non-cooperative patients and those undergoing complex orthodontic mechanics—such as intrusion arches, vertical elastics for open-bite correction, or severe dental torque (e.g., Class II Division 2 cases)—were excluded. Periodontal disease was also an explicit exclusion criterion; all enrolled participants had a clinically healthy periodontium confirmed at baseline examination. This criterion was strictly applied across both groups to eliminate periodontal inflammation as a local confounding factor for salivary biomarker levels.

To ensure comparability with the case group in cumulative mechanical exposure, control participants had to complete at least 36 months of active fixed appliance therapy, with a mean treatment duration of 39 months.

#### Radiographic assessment

Root resorption assessment was performed at a single cross-sectional time point, either during active orthodontic treatment after at least 10 months of therapy or at treatment completion. Imaging was based primarily on panoramic radiographs obtained as part of routine clinical care. Additional periapical radiographs were obtained only when panoramic findings indicated possible root resorption and greater diagnostic resolution was clinically warranted; no radiographs were taken exclusively for research purposes.

To reduce variability in the magnitude and direction of orthodontic forces, patients treated with complex mechanics, including intrusion arches, vertical elastics, or heavy torque prescriptions, were excluded. Suspected root resorption cases were independently confirmed by an experienced orthodontist through comprehensive clinical and radiographic assessment. Root resorption severity was graded according to the Levander–Malmgren classification [12]:

- Grade I: Irregular or serrated root surface.
- Grade II: Root resorption of less than 2 mm.
- Grade III: Root resorption ranging from 2 mm to one-third of the root length.
- Grade IV: Root resorption exceeding one-third of the root length.

Only patients with Grade II or higher root resorption were included in the case group.

In accordance with the ALARA (As Low As Reasonably Achievable) radiation-protection principle, periapical radiographs were not obtained from control participants who had no clinical suspicion of pathology [20]. Restricting the OIIRR group to patients with moderate-to-severe resorption improved diagnostic separation and reduced potential overlap from undetectable mild apical blunting. The assessment focused primarily on the maxillary and mandibular incisors.

All radiographs were independently evaluated by two calibrated orthodontic examiners who were blinded to participant group allocation and biomarker concentrations.

#### Orthodontic treatment protocol standardization

All patients included in this study were treated in a single academic clinical setting following a standardized departmental orthodontic protocol. All cases used fixed orthodontic appliances with a 0.022-inch slot prescription, and treatment followed a uniform archwire sequence applied consistently across patients. Experienced orthodontists performed treatment planning and appliance placement within the same clinical unit to minimize operator-related variability. Although precise quantification of orthodontic force magnitude and direction was not feasible because the clinical records were retrospective, identical appliance systems and standardized mechanics were intended to reduce interpatient variability in biomechanical loading during orthodontic tooth movement. Matching for age and sex, use of a standardized orthodontic appliance system, exclusion of complex biomechanics, and uniform sample collection protocols were implemented to minimize selection bias and confounding.

#### Baseline cephalometric measurements

Baseline cephalometric measurements, including SN–MP and FMA, were obtained before the initiation of orthodontic treatment to assess each patient’s vertical skeletal growth pattern. The study included only patients whose baseline measurements fell within established normative limits (as defined by Steiner, Tweed, and Proffit). Patients with vertical or horizontal growth tendencies outside these normative ranges were excluded to minimize potential confounding effects on skeletal growth patterns. Statistical analysis confirmed no significant differences in baseline cephalometric measurements between the case and control groups, ensuring both groups were comparable in vertical skeletal pattern, which influences susceptibility to root resorption. These variables were selected a priori because vertical skeletal pattern and treatment duration are established risk modifiers for orthodontically induced root resorption.

#### Treatment duration

The total treatment duration was recorded for all patients included in the study. In the control group, the mean treatment time was 39 months, and no patient received treatment for less than 36 months. This ensured treatment duration was comparable to the case group, and the average treatment time was consistent with standard orthodontic protocols. Including treatment duration data helps account for this important variable, as it influences the risk of orthodontically induced root resorption.

#### Sample collection

Following informed consent, demographic and clinical information was collected using standardized questionnaires. Saliva samples were collected under controlled conditions in accordance with established guidelines for salivary research. All saliva samples were collected in a clinical setting between 09:00 and 11:00 AM to minimize diurnal variation in salivary biomarkers [21–23]. To further reduce flow-related and circadian variability, specimens were obtained using a standardized 5-min unstimulated passive drool protocol after a 1-h fast.

Participants were instructed to refrain from eating, drinking, consuming alcohol, caffeine, or nicotine, chewing gum (1-h fasting), or performing oral hygiene procedures for at least 60 min before sample collection [22, 24].

Upon arrival, participants rinsed their mouths gently with deionized water and rested for 10 min before sample collection [24]. Unstimulated whole saliva was collected using the passive drool method [24]. Participants were seated comfortably and instructed to allow saliva to pool naturally in the floor of the mouth, then to drool into a pre-chilled, labeled 50 mL polypropylene conical tube kept on ice. To reduce potential contamination from transient oral debris, saliva produced during the first minute was discarded. Saliva secreted over the subsequent 5-min period was collected as the analytical sample.

Collected saliva was deposited into labeled 50 mL Falcon tubes and immediately placed on ice packs to inhibit protease activity and prevent protein degradation [25]. A standardized protocol ensured that all samples were transported to the laboratory on ice packs within 120 min of collection [25]. Subsequently, centrifugation was carried out at 12,000×*g *for 15 min at 4 °C to pellet debris and mucin [26]. The resulting supernatant was aliquoted into 1.5 mL microtubes and stored at − 80 °C pending ELISA analysis.

All procedures were performed at Hamadan University of Medical Sciences between 2023 and 2025. The demographic profiles for both the root resorption and control groups are provided in S1.

#### ELISA assay

Saliva samples were analyzed using an enzyme-linked immunosorbent assay (ELISA) to quantify specific biomarkers implicated in root resorption, as identified through prior bioinformatics analyses. Commercially available, validated ELISA kits were employed to ensure analytical specificity and reproducibility, and all procedures were performed strictly in accordance with the manufacturers' instructions under standardized laboratory conditions. To minimize potential bias, laboratory personnel conducting the ELISA assays were blinded to the clinical group allocation of the samples.

Salivary Caspase-1 concentrations were measured using a human Caspase-1 ELISA kit (Cat. No. DCA100, R&D Systems, USA) [27], which utilizes validated antibodies and has a manufacturer-reported analytical sensitivity of approximately 1.24 pg/mL, with a detection range specified in the accompanying datasheet. Salivary Osteopontin levels were determined using a human Osteopontin ELISA kit (Cat. No. EH0248, FineTest, China) [28], with a reported analytical sensitivity of approximately 0.094 ng/mL, as stated by the manufacturer (https://www.fn-test.com/product/eh0248/). For each assay, standard curves were generated using a four-parameter logistic (4PL) regression model, and sample concentrations were calculated accordingly (S2).

#### Statistical analyses

Normality of distribution was confirmed using Shapiro–Wilk tests (all *p* > 0.05). Independent two-sample t-tests (assuming equal variances) were used to compare biomarker levels between groups. Effect sizes were calculated using Cohen’s *d* and interpreted as small (0.2), medium (0.5), and large (≥ 0.8). Given the exploratory nature of this study and the independent biological pathways of the two biomarkers, each was analyzed separately without multiple comparison correction.

Receiver operating characteristic (ROC) curves were generated using the marker protein concentration data. This analysis evaluated the diagnostic potential of these markers for root resorption. Optimal cutoff values were determined by maximizing the Youden Index (J = sensitivity + specificity—1), which ranges from 0 to 1, with higher values indicating better overall diagnostic performance. All ROC curve analyses, including the calculation of area under the curve (AUC) and corresponding p-values, were conducted using GraphPad Prism software version 8.4.1.671.

Effect sizes were calculated using Cohen's *d* to quantify the magnitude of group differences. A post hoc power analysis was performed using G*Power 3.1 based on the observed effect sizes, with α = 0.05.

## Results

### PPI network analysis

Candidate biomarkers associated with root resorption were identified using two complementary approaches: a structured literature review and interrogation of the DisGeNET database. The literature review and gene curation processes were conducted independently by two authors. The authors resolved discrepancies in gene inclusion through discussion and consensus. This approach enhanced consistency and minimized selection bias during data extraction. Given the exploratory nature of the bioinformatics analysis, no formal inter-rater reliability metrics were calculated. The literature review yielded 133 genes reported to be associated with root resorption. In parallel, 43 root resorption-related genes were retrieved from the DisGeNET database using the disease identifier C0035851. The gene sets obtained from both sources were merged, and duplicate entries were removed. After deduplication, 160 unique genes were retained and used to construct a PPI Network (S3).

**Fig. 2:**
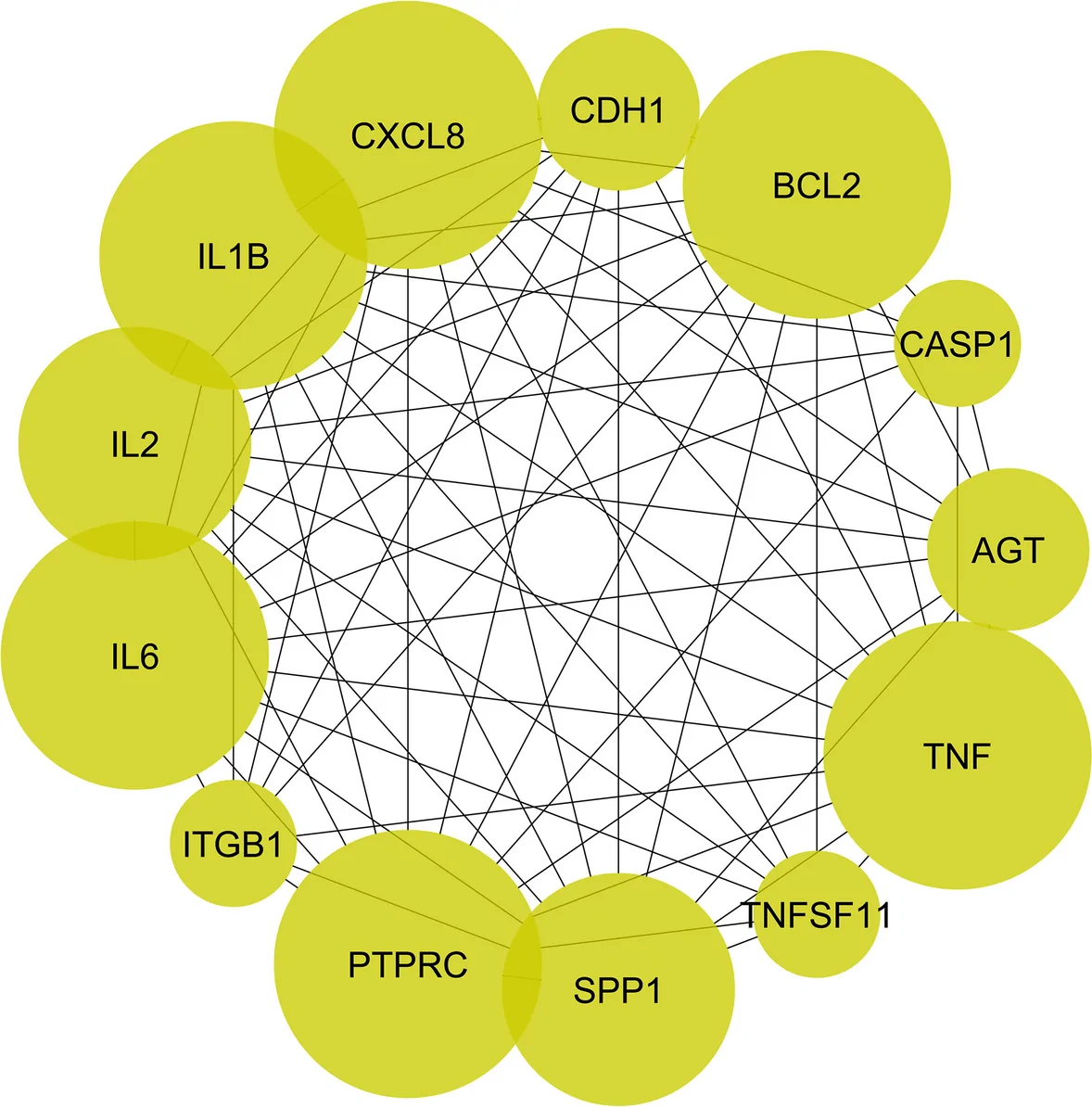
Internal interactions among 13 hub genes in the PPI network are associated with root resorption pathogenesis. Node size correlates positively with gene degree within the network. PPI, protein–protein interaction

**Fig. 3:**
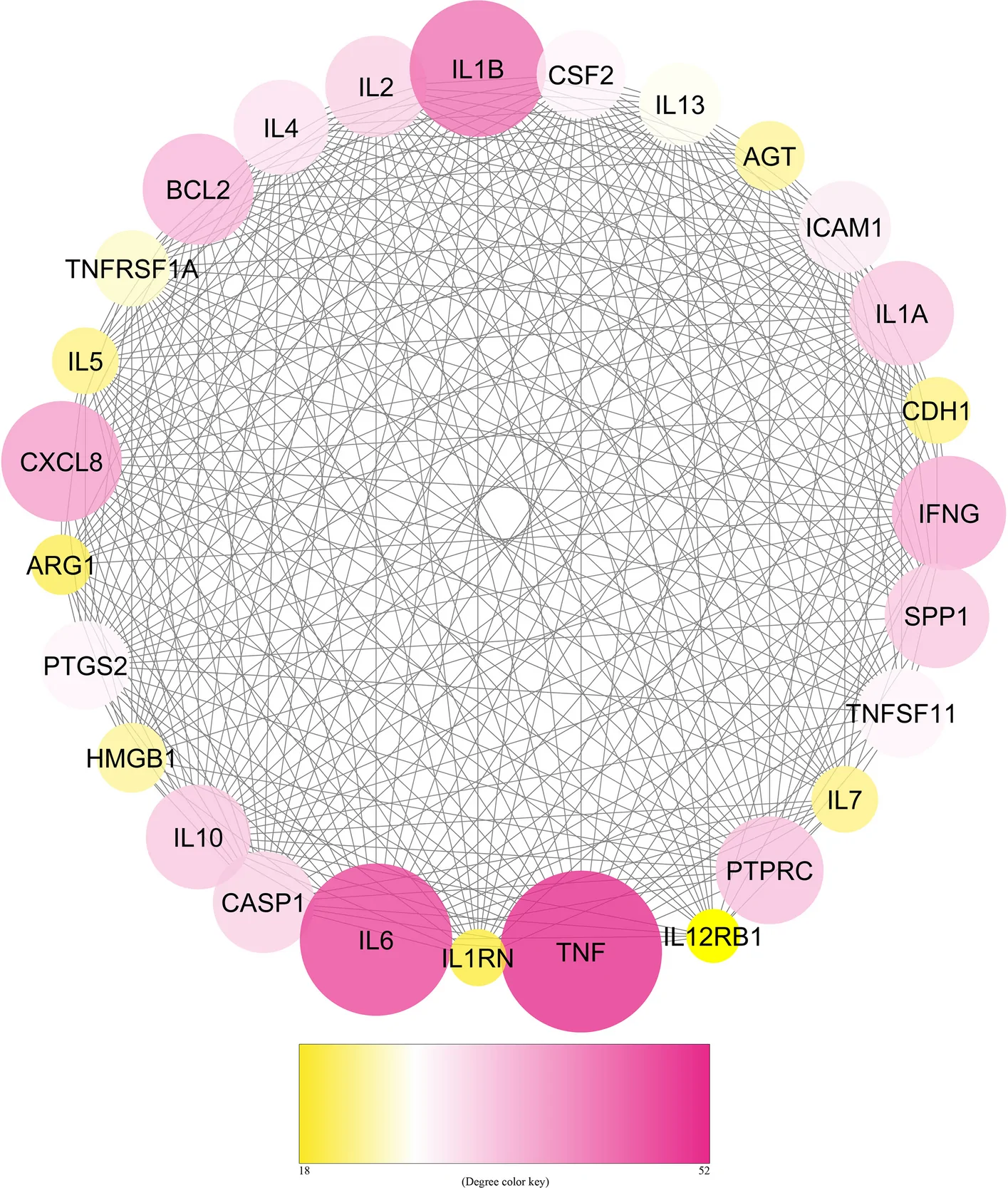
MCODE cluster within the root resorption PPI Network. Node size and color intensity (darker red for higher degree, darker yellow for lower) correlate positively with gene degree. MCODE, molecular complex detection complex; PPI, protein–protein interaction

The PPI network was constructed using the STRING database. Interactions were retrieved using a confidence score threshold of 0.4, corresponding to medium-confidence interactions as defined by STRING. This threshold balanced interaction reliability with network completeness, allowing the inclusion of experimentally supported and computationally predicted interactions relevant to the biological context of root resorption. Isolated nodes were subsequently removed from the network, yielding a Cytoscape graph with 120 nodes and 648 edges. Through structural analysis, 13 genes were identified as potential hub proteins within the root resorption PPI Network (Table 1), as their degree and betweenness values exceeded twice the mean, and their closeness values exceeded the average node feature. Average values of 10.8 for degree centrality, 0.01595 for betweenness centrality, and 0.3658 for closeness centrality were calculated. Figure 2 demonstrates the internal interactions among hub genes. In addition, one module was identified within the PPIN, comprising more than ten genes and an MCODE score exceeding three. This cluster included 27 genes and 304 edges with an MCODE score of 23.385 (Fig. 3).

**Table 1:** Root resorption hub genes from PPI network analysis (*n* = 13), ranked by degree

Name	Degree	Betweenness	Closeness
*TNF*	52	0.196	0.567
*IL6*	49	0.105	0.536
*IL1B*	44	0.054	0.524
*CXCL8*	39	0.045	0.509
*BCL2*	36	0.116	0.504
*PTPRC*	35	0.056	0.498
*SPP1*	34	0.058	0.484
*IL2*	33	0.051	0.478
*CASP1*	33	0.037	0.467
*TNFSF11*	29	0.032	0.461
*ITGB1*	23	0.063	0.461
*AGT*	23	0.060	0.451
*CDH1*	22	0.049	0.465

*PPI* protein–protein interaction

**Fig. 4:**
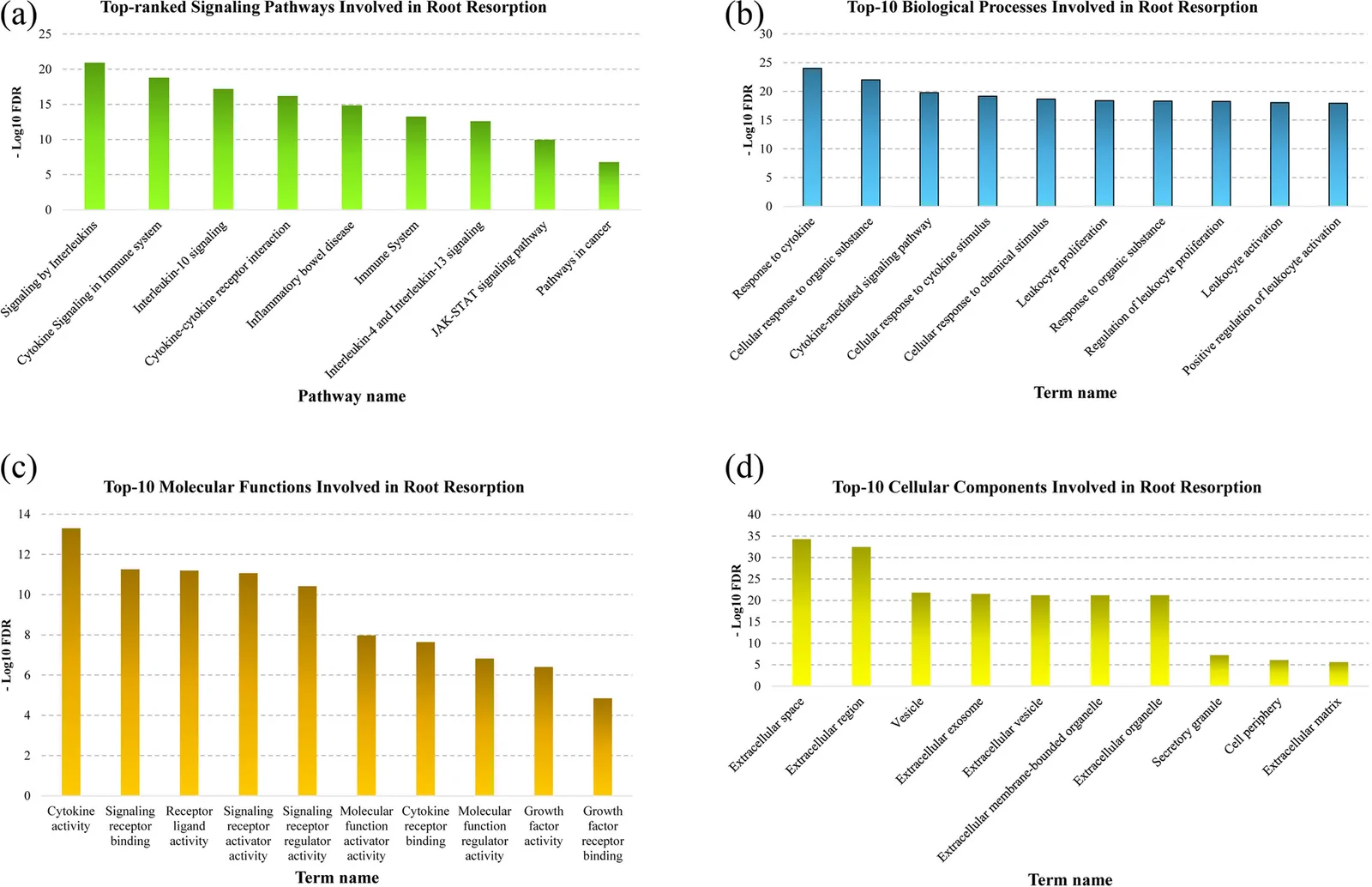
Top-ranked functional enrichments in root resorption. This figure shows the most affected pathways (**a**), biological processes (**b**), molecular functions (**c**), and cellular components (**d**). Only nine pathways met the significance criteria (false discovery rate < 0.05 and ≥ 10 genes)

Functional analysis of the identified cluster revealed pathways and biological processes significantly dysregulated in root resorption. As shown in Fig. 4a, b, the results suggest that several immune-related biological processes mediate root resorption. Specifically, the top 3 enriched pathways were “Signaling by interleukins,” “Cytokine signaling in immune system,” and “Interleukin-10 signaling.” Additionally, “Response to cytokine,” “Cellular response to organic substance,” and “Cytokine-mediated signaling pathway” were the top three biological processes associated with root resorption.

For the elucidation of MFs and CCs experiencing substantial perturbations in root resorption, 160 root resorption-related genes were submitted to g:Profiler. The analysis revealed that “Cytokine activity,” “Signaling receptor binding,” and “Receptor ligand activity” emerged as the most significantly enriched MFs in root resorption. Furthermore, “Extracellular space,” “Extracellular region,” and “Vesicle” were the principal cellular components showing significant dysregulation. Figures 4c, d depict the top-ranked affected MFs and CCs in root resorption.

### Upstream regulators in root resorption

**Fig. 5:**
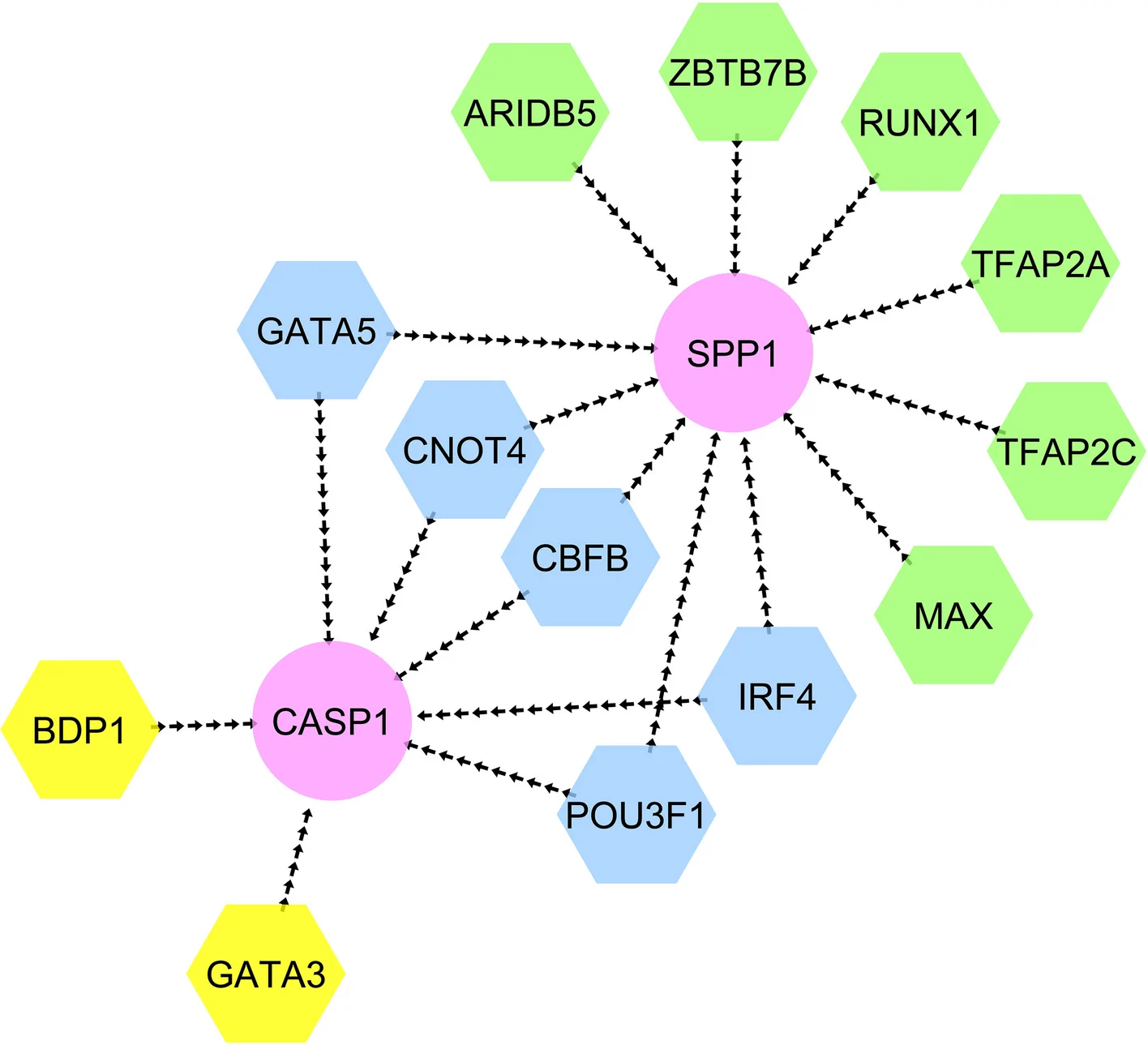
A Gene Regulatory Network of root resorption hub genes and their upstream regulators. Circles represent hub genes, while hexagons denote their regulators. Yellow and green hexagons indicate transcription factors (TFs) regulating *CASP1* and *SPP1*, respectively. Blue hexagons show TFs that affect both *CASP1* and *SPP1* expression. TF, transcription factor

From the 13 identified hub genes, *CASP1* and *SPP1* were selected for subsequent clinical analyses based on a combination of objective criteria. Both genes exhibited high topological significance within the PPI network. From a pathophysiological perspective, they are also critically involved in inflammation (Caspase-1) [28] and bone remodeling/osteoclastogenesis (Osteopontin) [29], which are primary mechanisms underlying OIIRR. In addition, to facilitate non-invasive clinical validation, we prioritized genes encoding secreted or reliably detectable proteins in human saliva [29, 30]. Therefore, a GRN was then constructed using the iRegulon plugin to identify putative upstream regulators of *CASP1* and *SPP1* expression (Fig. 5). Transcription factors (TFs) with an NES exceeding 10 were deemed significant. This analysis pinpointed 13 potential TFs for *SPP1* and/or *CASP1*, detailed in S4. Among TFs targeting both CASP1 and SPP1, IRF4 had the highest score (10.615).

### Salivary CASP1 and SPP1 expression

A total of 130 orthodontic patients were initially screened for eligibility. After applying inclusion and exclusion criteria, 24 patients were enrolled, comprising 12 cases and 12 matched controls. No participants were excluded after enrollment, and all samples were included in the final analysis. The protein expression levels of *CASP1* and *SPP1* in the saliva of patients with root resorption (case group) and patients with no signs of root resorption (control group) were examined using an ELISA assay. The ELISA used antibodies against Caspase-1 (Cat No: DCA100, R&D, USA) and Osteopontin (Cat No: EH0248, Finetest, China). Optical densities (ODs) were converted to protein concentrations via standard curves (S2).

### Statistics and diagnostic potential

The Shapiro–Wilk normality test was performed to assess data distribution. The results indicated a normal distribution for both *CASP1* and *SPP1* expression levels in both the case and control samples, with *p*-values exceeding 0.05 (*CASP1*_case: *p* = 0.8282; *CASP1*_control: *p *= 0.1933; *SPP1*_case: *p* = 0.977; *SPP1*_control: *p* = 0.597). Consequently, an independent-samples t-test was used to determine whether the average protein expression levels differed significantly between the groups. The *t*-test showed significantly higher CASP1 and SPP1 protein expression in the saliva of patients with root resorption than in the control group (*p* < 0.05). Specifically, *CASP1* expression in the case group (mean = 28.24550 ng/ml) was significantly higher than in the control group (mean = 13.24367 ng/ml; *p*-value = 2.193 × 10 − 7), representing a 2.13-fold increase. Similarly, *SPP1* expression was significantly higher in the case group (mean = 1.450083 ng/ml) than in the control group (mean = 1.024 ng/ml; *p*-value = 0.02862), corresponding to a 1.42-fold increase. Statistical analyses were conducted using R version 4.4.1.

**Fig. 6:**
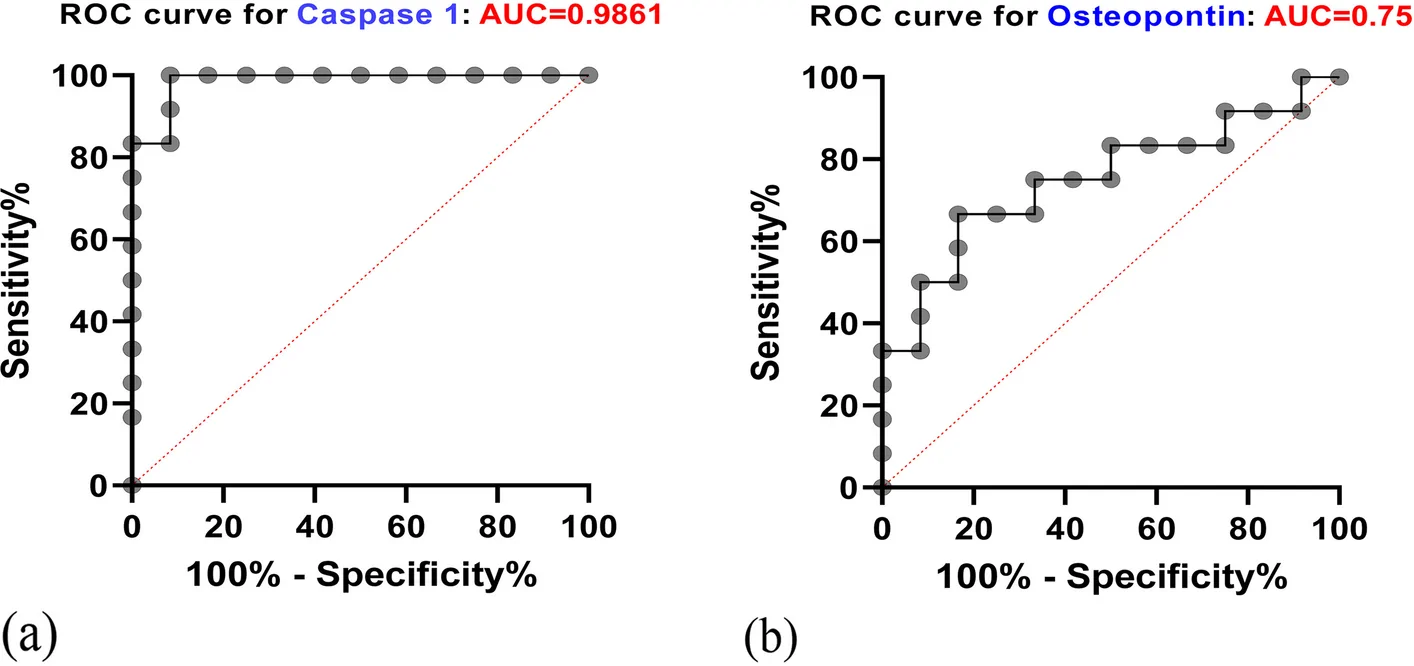
ROC curves for salivary *CASP1* and *SPP1* expression in the diagnosis of root resorption. **a** ROC curve for *CASP1*, demonstrating an AUC of 0.9861 (*p* < 0.0001), indicating excellent diagnostic accuracy. **b** ROC curve for *SPP1*, with an AUC of 0.75 (*p* < 0.05), suggesting its potential as a diagnostic marker. ROC, receiver operating characteristic; AUC, area under the curve

ROC curve analysis was performed to assess the diagnostic utility of salivary *CASP1* and *SPP1* for root resorption. Statistical analysis indicated a strong diagnostic potential for *CASP1*, with an AUC of 0.9861 (*p* < 0.0001). For *SPP1*, the AUC was calculated as 0.75 (*p* < 0.05), indicating its potential as a diagnostic marker as well (Fig. 6). Furthermore, the optimal cutoff values determined using GraphPad Prism were 18.75 ng/mL for *CASP1* and 1.224 ng/mL for *SPP1*.

Caspase-1 demonstrated excellent diagnostic accuracy with an AUC of 0.986 (95% CI 0.953–1.000). The in-sample optimal cutoff of 18.75 yielded 100% sensitivity, 91.67% specificity, and a Youden Index of 0.917. Osteopontin showed moderate diagnostic utility with an AUC of 0.75 (95% CI 0.543–0.957). At the in-sample optimal cutoff of 1.244, sensitivity was 66.67%, specificity was 83.33%, and the Youden Index was 0.50. Furthermore, Cohen’s *d* values of 3.01 for Caspase-1 and 0.96 for Osteopontin indicated large effect sizes, especially for Caspase-1. A post hoc power analysis revealed an achieved power of approximately 99% for Caspase-1 and 78% for Osteopontin, based on the observed effect sizes.

Given the pilot sample size and the absence of independent test datasets, these performance metrics and threshold values reflect exploratory discrimination within the current study group. They should not be interpreted as definitive diagnostic criteria.

## Discussion

This study underscores the significant role of 13 hub genes, including *CASP1* and *SPP1*, in the pathogenesis of root resorption. Furthermore, the results demonstrate a pronounced enrichment of immune-related pathways and BPs underlying this condition. Network-based bioinformatics analysis identified *IRF4* as a putative regulatory gene within the GRN, influencing the expression of *CASP1* and *SPP1* and suggesting an association with the etiology of root resorption. However, this inference is based on computational predictions and warrants experimental validation in future studies.

Identifying *Caspase-1* as a putative central hub gene in this study, along with its significantly increased abundance in the saliva of patients with root resorption, supports a potentially important role in disease pathogenesis. Rather than representing a nonspecific consequence of inflammation, emerging mechanistic evidence indicates that Caspase-1 may directly promote root resorption through defined cellular pathways. For example, under excessive mechanical loading, Caspase-1 induces pyroptosis in periodontal ligament stem cells (PDLSCs). This Caspase-1-dependent process alters interactions between PDLSCs and osteoclast precursors by promoting the transfer of damaged mitochondria and elevating reactive oxygen species (ROS) levels in precursor cells. These changes ultimately increase osteoclast abundance and activity, contributing to hard-tissue loss [31]. Therefore, Caspase-1 may represent an upstream mediator of PDLSC pyroptosis and the subsequent osteoclastogenesis underlying root resorption.

Moreover, the emergence of *SPP1*, which encodes Osteopontin, as a key hub gene in this study offers a mechanistic explanation for the resorptive changes observed clinically. In orthodontic loading, where periodontal tissues experience both tensile and compressive forces, Osteopontin is not simply an inflammatory marker or bystander molecule. Besides, it acts as an important regulator of periodontal tissue remodeling. Osteopontin can promote hard-tissue degradation by stimulating osteoclast differentiation and supporting osteoclast survival and migration through CD44- and αvβ3-dependent signaling. It also contributes to osteoclast podosome formation, which is essential for bone and root resorption, while suppressing osteoblast mineralization through a phosphate-dependent mechanism [32].

Several studies have investigated the role of *CASP1* in root resorption. Zhang et al. [33] used a rat model to investigate the roles of the NOD-like receptor protein 3 (*NLRP3*) inflammasome and M1 macrophages. They observed that *NLRP3*, *CASP1*, *IL-1β*, and M1 macrophages were present in the periodontal ligament, primarily in areas of root resorption. In vitro experiments using a co-culture of macrophages and human periodontal ligament cells (hPDLCs) revealed that hPDLCs pre-treated with force promoted M1 macrophage polarization and increased *NLRP3*, *CASP1*, and *IL-1β* production in M1 macrophages. Inhibiting *NLRP3* inflammasome activation with MCC950 reduced both *NLRP3* activation and M1 macrophage polarization. These findings suggest that NLRP3 inflammasome and CASP1 activation in M1 macrophages initiate root resorption.

Baghaei et al. [34] conducted a case-control study to assess the impact of specific SNPs and other factors on EARR (external apical root resorption) in orthodontic patients. They found significant associations between root resorption and both the extraction treatment protocol and dental classification. The study also noted that the GG genotype of the *IL-1A* gene variant increased susceptibility to EARR in Caucasians. Although the study observed a potential association between a CASP1 gene variant and root resorption, it did not reach statistical significance after adjustment. These results indicate that a combination of patient- and treatment-related factors, as well as genetic variations, influences EARR.

Nieto-Nieto et al. [35] reviewed the genetic predispositions to EARR during orthodontic treatment. They highlighted the influence of various genetic biomarkers, including genes involved in the inflammatory response (*IL1B*, *IL1A*, *IL1RN*, *IL6*), purinergic signaling (*P2RX7*), and apoptosis (*CASP1*). Their review underscored the complex interplay between genetic factors, mechanical forces from orthodontic treatment, and environmental factors in the development of EARR. The authors emphasized the need for further research to fully clarify the precise nature of EARR and the extent of genetic inheritance's influence.

Iglesias-Linares et al. [36] explored the connection between the *SPP1* gene and EARR in orthodontic patients. Their study involved 87 individuals undergoing orthodontic treatment and examined two polymorphisms within the *SPP1* gene cluster (rs9138 and rs11730582). The findings indicated that patients who were heterozygous or homozygous for the most common allele at *SPP1* positions 89,261,521 and 89,253,600 had a protective effect against post-orthodontic EARR. Conversely, individuals homozygous for SPP1 at rs9138 and rs11730582 showed an elevated risk of developing EARR.

Jimenez-Pellegrin et al. [37] investigated the repair of root resorption during orthodontic rotation. They utilized transmission electron microscopy and *SPP1* immunolabeling to analyze the repair process within resorbed cementum. Their results revealed evidence of repair in previously resorbed lacunae, with both clastic cells and the root surface showing *SPP1* immunolabeling. The authors concluded that cementum repair occurs after resorption during rotational tooth movement and that *SPP1* participates in both resorption and repair.

The combined findings of Iglesias-Linares et al. [36] and Jimenez-Pellegrin et al. [37] strongly suggest that *SPP1* plays a critical role in root resorption. Genetic variations in SPP1 appear to influence an individual’s susceptibility to, or protection against, EARR in orthodontic patients. Moreover, *SPP1* has been implicated in repairing resorbed cementum during orthodontic tooth movement. These insights underscore the significant impact *SPP1* may have on both the progression and resolution of root resorption.

Gene set enrichment analysis revealed significant enrichment of immune system-related pathways in root resorption, with “Signaling by interleukins,” “Cytokine signaling in immune system,” and “Interleukin-10 signaling” emerging as the top three dysregulated pathways. The prominence of biological processes, including “Response to cytokine,” “Cellular response to organic substance,” and “Cytokine-mediated signaling pathway,” further underscores the central role of immune mechanisms in root resorption pathogenesis.

The available evidence suggests that root resorption is characterized by immune system dysregulation. This dysregulation manifests as an imbalanced inflammatory response where the standard regulatory mechanisms fail to maintain tissue homeostasis.

Multiple studies show elevated pro-resorptive cytokines in root resorption. Ahuja et al. [38] documented significant increases in *TNF-α* and *IL-7* in gingival crevicular fluid following heavy orthodontic forces, correlating with increased root resorption severity. Similarly, Nakano et al. [39] identified *IL-17* as a key mediator that directly upregulates *RANKL* expression in dental pulp cells, thereby promoting osteoclast activation and root resorption. These findings indicate that specific pro-inflammatory pathways are aberrantly activated during root resorption.

Importantly, the immune dysregulation in root resorption is not unidirectional. Ahuja et al. [38] observed significantly elevated levels of *CSF2* (granulocyte–macrophage colony-stimulating factor), an anti-resorptive cytokine, in cases with mild root resorption, suggesting that the immune system attempts to counterbalance pro-resorptive signals through compensatory anti-inflammatory mechanisms. This finding supports a model of dysregulated rather than hyperactivated immunity, in which both pro- and anti-inflammatory mediators are elevated but inadequately coordinated.

Seifi et al. [40] provided crucial mechanistic insights by demonstrating that orthodontically induced inflammatory root resorption arises from sterile necrosis in the periodontal ligament, driven by compromised vascularization. Their finding that basic fibroblast growth factor (*bFGF*) significantly reduced root resorption by promoting angiogenesis suggests that tissue hypoxia triggers sterile inflammatory responses that contribute to immune dysregulation. This hypoxia-induced inflammation represents a pathological activation of immune pathways in the absence of infectious agents.

The study by Nishioka et al. [41] revealed another dimension of immune involvement by showing that patients with constitutional immune alterations, specifically those with allergies and asthmatic tendencies, show increased susceptibility to excessive root resorption during orthodontic treatment. This finding suggests that baseline immune system characteristics influence the magnitude of dysregulated responses to mechanical stress, indicating that pre-existing immune system alterations may amplify immune dysregulation in root resorption.

While these predictive network findings highlight candidate pathways, functional experiments are needed to determine whether interventions that restore immune balance, rather than simple anti-inflammatory approaches, may be more effective at preventing or mitigating root resorption. Furthermore, identifying constitutional risk factors opens the door to personalized risk assessment and preventive strategies in orthodontic treatment planning.

Based on our computational inference, *IRF4* was predicted as a potential upstream regulator of *CASP1* and *SPP1*, with a notably high NES score. While the literature review focuses on *IRF8*’s role in root resorption, the distinct yet related functions of *IRF* family members should be acknowledged.

The provided study by Das et al. [42] clearly establishes *IRF8* as a crucial negative regulator of osteoclast activity and a key player in maintaining dental and skeletal homeostasis. Mutations in *IRF8*, specifically the *IRF8* G388S variant, have been linked to increased susceptibility to multiple idiopathic cervical root resorptions due to enhanced osteoclast formation and resorption. Both studies showed that impaired IRF8 function increases osteoclastogenesis by failing to inhibit *NFATC1*-dependent transcriptional activation and by affecting autophagy-related pathways. This highlights *IRF8’s* critical role in defining osteoclast lineage and its transcriptional program.

Although *IRF4* and *IRF8* are distinct, they both belong to the interferon regulatory factor family. While *IRF8* has been shown to regulate osteoclasts negatively, the specific role of *IRF4* in this context warrants further investigation. It is plausible that *IRF4*, like other *IRF* family members, could influence osteoclast activity, potentially through mechanisms related to, or distinct from, those described for *IRF8*. For instance, *IRF4* interacts with other transcription factors and co-regulators, potentially affecting the expression of genes such as *CASP1* and *SPP1*, which are implicated in root resorption. The significant NES score for *IRF4* in our GRN analysis suggests a strong potential for its involvement as an upstream regulator, warranting further functional studies to elucidate its precise role in root resorption and its interaction with the osteoclast differentiation pathway. Future research could investigate whether *IRF4* influences pathways similar to those influenced by *IRF8*, or whether it exerts its effects through alternative mechanisms that contribute to the pathogenesis of root resorption.

Identifying CASP1 and SPP1 as salivary biomarkers for orthodontic root resorption aligns with and extends findings from genetic association studies. A recent systematic review by Andrade et al. [43] on genetic polymorphisms in EARR across 21 studies reported that CASP1/ICE and SPP1 gene polymorphisms have been associated with root resorption susceptibility. However, results have been conflicting across populations. While genetic studies identified *IL1* family genes as most consistently associated with EARR and *P2RX7* as another important factor, individual studies showed variable associations with *CASP1* polymorphisms, with some finding significant associations and others reporting no effect. Our protein-level validation advances this field by demonstrating that, regardless of genetic polymorphism status, elevated salivary *CASP1* and *SPP1* protein expression serves as a functional biomarker of active root resorption, with *CASP1* showing exceptional diagnostic performance (AUC = 0.9861, Cohen’s *d* = 3.01). This distinction matters clinically: genetic testing identifies inherited susceptibility, while salivary protein biomarkers detect ongoing resorptive activity. The very large effect sizes observed in our study suggest that protein expression changes may be more pronounced and consistent than genetic associations, potentially explaining the variability in previous genetic studies across ethnic groups. Our pathway enrichment analysis corroborating immune system dysregulation—particularly “Signaling by interleukins” and “Cytokine signaling”—provides a mechanistic link between the genetic associations described by Andrade et al. [43] and the functional inflammatory processes driving root resorption, suggesting that integrating genetic susceptibility screening with protein biomarker monitoring may represent the optimal strategy for comprehensive EARR risk management.

Based on ELISA results analyzed with GraphPad Prism, the optimal diagnostic cutoffs were 18.75 ng/mL for *CASP1* and 1.224 ng/mL for *SPP1*. Protein concentrations exceeding these thresholds indicate root resorption, whereas levels below them suggest its absence. The present findings indicate meaningful translational potential, positioning *CASP1* as a relevant biomarker associated with both the development and resolution of root resorption [36, 37]. The elevated salivary Caspase-1 levels observed in orthodontic patients with root resorption, compared with those without, support its potential utility as a non-invasive indicator of pathological activity and treatment responsiveness. Clinically, quantifying salivary Caspase-1 may help with early risk stratification, enable longitudinal monitoring of disease progression, and provide an objective measure for evaluating interventions aimed at reducing tissue damage. In addition, Caspase-1 may represent a candidate therapeutic target for preventing root resorption in orthodontic patients, although this hypothesis requires further experimental and clinical validation. While larger longitudinal studies are needed before clinical implementation, these results provide a rationale for integrating biomarker-guided assessment into more personalized patient management strategies.

Importantly, the control group consisted of age- and sex-matched orthodontic patients undergoing active fixed-appliance treatment who had an intact, clinically healthy periodontium and no radiographic evidence of root resorption. Therefore, the significant increases in salivary CASP1 and SPP1 are unlikely to be explained solely by baseline orthodontic tooth movement or generalized mechanical stress. Instead, because both groups shared the physiological inflammatory response associated with active orthodontic treatment, the observed upregulation of CASP1 and SPP1 may reflect the specific cellular and osteoclastogenic processes involved in active OIIRR.

This in silico analysis should be regarded as exploratory because it relied exclusively on previously published literature and databases, including DisGeNET, rather than on de novo transcriptomic or proteomic profiling in the study population. This approach is susceptible to literature and annotation bias, which may favor well-characterized genes and underrepresent less-studied or previously unidentified regulators of root resorption. Moreover, network centrality indicates statistical connectivity rather than established biological causation; consequently, the inferred interactions may not reflect the processes occurring within the mechanically stressed periodontal microenvironment. The clinical validation was cross-sectional and involved only 12 patients with root resorption and 12 controls, supporting its classification as a preliminary pilot study. Because the study did not monitor biomarkers longitudinally during active orthodontic treatment, it could not establish a temporal relationship between biomarker changes and root resorption progression. The exceptionally high diagnostic performance observed for Caspase-1 (AUC = 0.9861) may therefore reflect the small sample size and should be interpreted cautiously until confirmed in larger populations. Caspase-1 and Osteopontin were analyzed independently without adjustment for multiple comparisons. Although this strategy is appropriate for hypothesis generation, confirmatory research should apply stricter procedures, such as the Bonferroni correction or FDR control. The absence of an independent external validation cohort also limits the immediate generalizability of the findings. Although regulatory networks were generated, evidence supporting therapeutic applications remains limited and theoretical; such implications require extensive validation in in vivo models. Translation into clinical practice poses further challenges because salivary protein concentrations may vary with collection time, salivary flow rate, and transient oral inflammatory conditions such as mild gingivitis. Standardized sampling procedures and affordable, rapid chairside assays will be necessary before these biomarkers can be used for real-time orthodontic monitoring. Large, prospective, multicenter studies are needed to replicate the findings, define robust diagnostic thresholds, and determine the clinical utility of these salivary biomarkers. In addition, the precise treatment stage at which root resorption first occurred could not be confirmed retrospectively from our dataset. Also, periapical radiographs were obtained selectively to confirm suspected cases of root resorption, whereas routine radiographic monitoring primarily relied on panoramic images.

## Conclusion

This study suggests 13 hub genes in OIIRR as potential candidate biomarkers and targets for future research. Targeted ELISA validation showed elevated salivary levels of Caspase-1 and Osteopontin in orthodontic patients with root resorption compared with unaffected controls. While initial evaluations suggest that Caspase-1 holds substantial promise as a non-invasive candidate biomarker, this exceptionally high diagnostic performance must be interpreted with caution given the limited sample size of the current cohort and may reflect an overestimation of clinical potential; it should be regarded as exploratory rather than conclusive evidence of diagnostic validity. Furthermore, the computational identification of *IRF4* as a predicted upstream regulator offers new perspectives on the regulatory networks governing *CASP1* and *SPP1* expression, supporting the hypothesis that immune system dysregulation contributes to the pathogenesis of the condition. While these findings highlight promising molecular targets and pathways, large-scale, prospective, multicenter studies are essential to validate their clinical diagnostic utility and therapeutic potential definitively.

## Supplementary Information

Supplementary Material 1: S1. Demographic characteristics of orthodontic patients: root resorption vs. non-root resorption groups.

Supplementary Material 2: S2. Representative standard curves for ELISA assays. (a) Caspase-1 was measured using a commercial human Caspase-1 ELISA kit (Cat. No. DCA100, R&D Systems, USA; analytical sensitivity ~ 1 pg/mL). (b) Osteopontin was measured using a commercial human Osteopontin ELISA kit (Cat. No. EH0248, FineTest, China; detection limit ~ 0.1 ng/mL). Both assays were quantified using a four-parameter logistic (4PL) fit to the standard curve data

Supplementary Material 3: S3. A comprehensive analysis combining findings from a systematic review and the DisGeNET database identified 160 genes associated with root resorption

Supplementary Material 4: S4. Thirteen transcription factors influence *CASP1* and/or *SPP1* expression.

## Data Availability

Data available on request due to privacy/ethical restrictions.
