# Supplementary material for: A systems biology network analysis of root resorption biomarkers and their clinical relevance in orthodontics: a pilot study
Source: Genomics Inform. 2026 Sep 28;24:16. doi: 10.1186/s44342-026-00079-2 (PMC13617904; doi:10.1186/s44342-026-00079-2)
Supplement: Supplementary file 1 [file 44342_2026_79_MOESM1_ESM.docx]

**Supplementary Data 1.** Demographic characteristics of orthodontic patients: root resorption vs. non-root resorption groups.

| **Demographic characteristics** | **Root resorption (Case)** | **Non-root resorption (Control)** |
| --- | --- | --- |
| Average age of orthodontic patients, years | 22.17 | 22.67 |
| Female/male ratio in volunteers | 9/3 | 11/1 |
