# Supplementary material for: A systems biology network analysis of root resorption biomarkers and their clinical relevance in orthodontics: a pilot study
Source: Genomics Inform. 2026 Sep 28;24:16. doi: 10.1186/s44342-026-00079-2 (PMC13617904; doi:10.1186/s44342-026-00079-2)
Supplement: Supplementary file 2 [file 44342_2026_79_MOESM4_ESM.docx]

**Supplementary Data 4.** Thirteen transcription factors influencing *CASP1* and/or *SPP1* expression.

| **TF** | **Target(s)** | **NES** |
| --- | --- | --- |
| *TFAP2A* | *SPP1* | 16.091 |
| *TFAP2C* | *SPP1* | 14.55 |
| *MAX* | *SPP1* | 12.695 |
| *BDP1* | *CASP1* | 12.153 |
| *GATA3* | *CASP1* | 10.755 |
| *ZBTB7B* | *SPP1* | 10.655 |
| *RUNX1* | *SPP1* | 10.648 |
| *IRF4* | *CASP1, SPP1* | 10.615 |
| *CNOT4* | *CASP1, SPP1* | 10.532 |
| *POU3F1* | *CASP1, SPP1* | 10.383 |
| *GATA5* | *CASP1, SPP1* | 10.267 |
| *CBFB* | *CASP1, SPP1* | 10.052 |
| *ARIDB5* | *SPP1* | 10.018 |

NES, normalized enrichment score.
